# The representation of migrants in policy and parliament: A Bacchian analysis of the UK's immigration health surcharge

**DOI:** 10.1177/02610183251386386

**Published:** 2025-11-03

**Authors:** JOANNE ALEXANDER, AISHA MACGREGOR, LAURENCE LESSARD-PHILLIPS, TIM SEDGLEY, LIZ FORBAT

**Affiliations:** 7622 University of Stirling, UK; 7622 University of Stirling, UK; 1724 University of Birmingham, UK; 1725 University of Stirling, UK; 7622 University of Stirling, UK

**Keywords:** Bacchi, immigration, health, problem representation

## Abstract

Despite the promise of the NHS being open to all, charging regulations and policy for non-UK citizens have been introduced. This article reports an analysis of policies and parliamentary debates linked to the UK's Immigration Health Surcharge. We use Bacchi's ‘what's the problem represented to be’ approach to understand how migrants and their healthcare access are represented and problematised within current health policy and related parliamentary debates. Core problem formulations relate to historic over-generosity of the NHS to migrants and overseas visitors; a lack of fairness in contributions to the NHS by British taxpayers compared to migrants; and a threat to the NHS's long-term sustainability due to migrants’ and overseas visitors’ misuse. This represents migrants as a financial drain on the NHS and, consequently, a risk to its continuation. Together, the problem formulations produce a justification and rationale for the Immigration Health Surcharge and its subsequent increases.

## Introduction

Immigration in the UK is a highly contentious issue with particularly intensive political emphasis on control in the last decade (Griffiths and Trebilcock, 2023). Immigration debates have spilled into a range of policy areas, including healthcare. This has taken place amidst a backdrop of austerity, heated debates about the strain on services, and a narrative of overseas visitors abusing the NHS (McHale and Speakman, 2020). Indeed, the implementation of policies that place limitations on access to publicly funded healthcare are now embedded within healthcare practice in many minority world countries (Grit et al., 2012; Hacker et al., 2015).

Despite the promise of the NHS being open to all, residence-based charging regulations and a charging policy were implemented under Thatcher's Conservative government in the 1980s (McHale and Speakman, 2020; Worthing et al., 2021). Following duress from stakeholders including migrant organisations, trade unions, law centres and health practitioners, the 1980s charge was discontinued (New Economics Foundation, 2020), but continued in different forms (McHale and Speakman, 2020). In 2015, the Immigration Health Surcharge (IHS) was introduced, considered the strictest approach yet (McHale and Speakman, 2020).

The IHS requires migrants and their dependents applying for specific visas (e.g., to work, to study) to pay an annual fee to allow use of the NHS without any additional charges for the duration of the visa terms (Gov.UK., 2023). The IHS does not explicitly define migrant, but the legislation (2015a) indicates that the policy refers to people holding visas, who are not ordinarily resident in the UK. What is covered by the IHS and what is free to anyone, however, is not simple (Department of Health and Social Care, 2024). In England, some services are without charge, regardless of status, including (but not limited to): seeing a GP, accident and emergency services, communicable disease services, and some palliative care services (Gov.UK., 2023).

Since the advent of the IHS, the annual charge has seen regular increases, from £150 per person in 2015 to £776-£1,035, depending on visa type (with health and care staff exempt) in 2024 (Immigration Health Order and Amendment, 2015a, 2015b). Given the continued problematisation of immigration, persistent annual fee increases suggest a strengthening government mandate and public opinion on immigration control (Griffiths and Trebilcock, 2023; Grit et al., 2012).

Implementation of charging has been seen by many as a mechanism to cultivate and perpetuate a hostile environment for migrants (Bowling and Westenra, 2020; Griffiths and Trebilcock, 2023; McHale and Speakman, 2020; Medact, 2020; Usborne, 2018). This links to a strategy and series of policies first introduced in 2012 by Theresa May in her role as Home Secretary which aimed to ‘create here in Britain a really hostile environment for illegal migration’ (Essex et al., 2022; Grierson, 2018; Worthing et al., 2021). The explicit articulation of a hostile environment speaks to the anti-immigrant context in which discrimination and marginalisation not only operate unrestrained, but actually functions ‘as an aspect of the good management of migration’ (Bowling and Westenra, 2020: 178).

It has been argued that the NHS has become a site of immigration control and bordering, helped by the IHS and associated identification and monitoring processes which grant or deny use (Reynolds and Mitchell, 2019; Worthing et al., 2021) and which enable the NHS to conduct status checks and share patient data with the Home Office (Department of Health and Social Care, 2019). Even in its early days, the IHS was linked closely to welfare chauvinism (Guentner et al., 2016). The IHS is part of the control of both migrant entry and stay and, it has been argued, that the charge acts to promote high skilled/high income migrants through its prohibitive costs, the so-called ‘good’ type of migration (Griffiths and Trebilcock, 2023). Hence, the IHS operates as a disciplinary mechanism for lower income, legal migrants intended to make their lives economically more uncomfortable.

Our research aims to critically analyse immigration health policy documents and parliamentary debates related to charging migrants to access healthcare. The analysis sought to better understand the representation of migrants in healthcare policy, and was nested in a wider project looking at the experiences of terminally ill migrants and their experiences of financial insecurity. The study incorporates desk reviews (a systematic review of terminally ill first generation migrants, analysis of palliative care policy, a media review) with qualitative interviews with health/social care/policy/legal professionals. The immigration health surcharge is a thread running throughout the datasets, forming a backdrop of access to the National Health Service. The central research question reported here is: What is the problem representation of migrants in Immigration Health Surcharge policy documents and parliamentary debates?

## Methodology

We conducted a critical analysis of policy documents and parliamentary debates related to the Immigration Health Surcharge (IHS). We drew on Bacchi's analytical approach, ‘what's the problem represented to be?’(Bacchi, 2009; Bacchi and Goodwin, 2016), known as ‘WPR’. This approach was chosen as it displaces assumptions that policies *solve* problems and asks how problems are represented in policy (Bacchi, 2009). This has an ontological fit with the research team who do not see migrants themselves as a problem to be solved. WPR enabled an interrogation of how immigration health policies and debates represent migrants and migrants’ access to and utilisation of healthcare systems. Informed by Foucault's writing on power, Bacchi's approach is explicitly focused on challenging and dismantling harmful representations including those which are minoritizing or marginalising. WPR analysis is focused on epistemology and ontology – specifically identifying and exploring the nature of problem construction and formulation within policy.

WPR analysis seeks to examine the ways ‘policy solutions are constituted by the assumptions entailed in the problematising process, rather than being self-evidently responsive to objective social ‘problems’’ (Bletsas and Beasley, 2012: 2). WPR is intended to act as a structure to critically interrogate policies, and views ‘problems’ and solutions as performative, constructive and constitutive. Policies are not solely a response to solve problems, but by their very nature constructed to articulate and define problems. Policies therefore ‘produce problems’ (Bacchi, 2009) and subjects, objects and places (Bacchi, 2022); the work of the WPR analysis is to identify the assumptions and logics implicit within the problem construction in policy.

## Data sources

Policy documents were defined as documents that articulate a set of ideas or plans which set expectations and boundaries about a course of action on the focal topic. We also included parliamentary debates into our analyses, given their central role in policy making (Proksch and Slapin, 2015).

To find relevant policy documents regarding the IHS, searches were conducted in Google, Gov.UK, NICE, and the House of Commons online library. Search terms are shown in the Appendix. The searches covered the 2010–2024 period (from the election of the Coalition government), to capture the policy context set by the Immigration Acts of 2014 and 2016, and the 2015 introduction of the IHS. The searches yielded 16 relevant documents. Three members of the team independently reviewed each of these and identified those most relevant to the research, with the study's Expert Advisory Group, we identified three interconnected policy documents (plus amendments) to include in the analysis (see Table 1). The selected policy documents include legislative documents around the setting of the IHS (the original Immigration (Health Charge) Order 2015 and subsequent amendments, as well as the Government's response to the 2017 public consultation about NHS charging and a House of Commons research briefing detailing the background and current status of the IHS.

**Table 1.** Included policy documents.

Document	Date	Producing agency	Intended audience	Jurisdiction
The Immigration (Health Charge) Order 2015(2015a)	March-April 2015 No.792	Government	Legislators	UK
The Immigration (Health Charge) Order 2015 – Subsequent Amendments (2015b)	2016 2017 2018 2020 2023 2024	Government	Legislators	UK
Making a fair contribution – Government response to the consultation on the extension of charging overseas visitors and migrants using the NHS in England (Department of Health, 2017)	06 Feb 2017	Department of Health	NHS-staff; NHS commissioners; patients & public; 3^rd^ sector representing vulnerable groups	England
The Immigration Health Surcharge (Research Briefing)	08 Nov 2023	House of Commons Library (Gower & McKinney)	MPs	UK

Parliamentary debates were extracted from the Hansard database, which retains verbatim reports of UK parliamentary discussions and decisions made by members of the House of Commons (HC) and the House of Lords (HL). Searches were conducted in the Hansard Database by two researchers, using combinations of key terms and phrases, such as immigration, immigrant, migration, migrant, asylum, refugee, immigration health charge, and immigration health surcharge. Searches were filtered to mirror the timeframe covered for the selection of policy documents and include debates between 1 January 2010 – 30 May 2024.

The phrase ‘immigration health surcharge’ yielded two debates (comprising 13 contributions, including a verbal interjection or turn taken within the debate), and ‘immigration health charge’ generated 12 debates (made up of 296 contributions). A relevance check was conducted for each of the debates by two researchers who deemed all 14 relevant for inclusion within our analysis. Debates involved contributors from a range of parties. Table 2 lists the parliamentary debates covered.

**Table 2.** Included parliamentary debates.

Title of debate	Date of debate	Producing agency	Volume	Contributors
Immigration health surcharge	13 March 2024	UK Parliament, HC	747	SNP - 1
Draft Immigration (Health Charge) (amendment) Order 2023	10 January 2024	UK Parliament, HC	Not stated	Cons – 10 Labour – 6 Labour and Co-op – 1
Immigration (Health Charge) (amendment) Order 2023	12 December 2023	UK Parliament, HL	Not stated	Cons – 1 Lib Dem – 1 Labour 1
Immigration (Health Charge) (Amendment) Order 2020	23 September 2020	UK Parliament, HL	805	Cons – 2 Lab −2 Non-affiliated – 1 LD −1
Draft Immigration (Health Charge) (Amendment) Order 2020	22 September 2020	UK Parliament, HC	Not stated	Cons – 9 Labour – 5 LD – 1 SNP - 1
Immigration health surcharge exemption	13 July 2020	UK Parliament, HC	678	Labour – 5 Cons - 1
Immigration (Health Charge) (Amendment) Order 2018	28 November 2018	UK Parliament, HL	794	Cons – 4 Labour – 4 LD – 2 Green – 1 Crossbench – 2 Non-affiliated - 1
Draft Immigration (Health Charge) (amendment) Order 2018	13 November 2018	UK Parliament, HC	Not stated	Cons – 10 Lab – 6 Lab/co-op – 1 LD – 1 SNP – 1
Draft Immigration (Health Charge) (amendment) Order 2017	9 Feb 2017	UK Parliament, HL	778	Cons – 1 Labour – 1 LD - 1
Draft Immigration (Health Charge) (Amendment) Order 2017	31 January 2017	UK Parliament, HC		Cons – 11 Lab – 6 SNP - 2
Immigration (Health Charge) (amendment) Order 2016	2 March 2016	UK Parliament, HL	769	Cons – 1 Lab −1
Draft Immigration (Health Charge) (Amendment) Order 2016	29 Feb 2016	Third Delegated Legislation Committee		SNP – 2 Cons – 10 Lab – 5 Lab/co-op – 2
Immigration (Health Charge) Order 2015	16 March 2015	UK Parliament, HL	760	Cons – 1
Immigration (Health Charge) Order 2015	10 March 2015	UK Parliament, HL	760	Cons – 2 Lab −1

*HL = House of Lords; HC = House of Commons.

## Analysis

We used Bacchi's WPR seven-step process to understand the problem definition which frames our analysis of relevant policy and parliamentary debates. First, we considered what ‘problems’ are identified within the policy and how they are constructed. Second, we examined taken for granted assumptions and deep-seated views underpinning the ‘problem’ being represented. Third we considered the historic context and genealogical lineage through which the representation of the ‘problem’ may have evolved and emerged from. Fourth, we explored what was voiced (and by whom), what was silenced and what was unproblematic within the policy account or debate and its problem formulation. Fifth we looked at the potential implications of the policy account and its problem representations including those who serve to benefit, those who become disadvantaged by it, those who are marginalised, and those who may be subject to its associated discourses and narratives. Sixth, we tracked how the problem representation continues to influence discourse related to the themes of the policy/debate. The final step calls for reflexivity from the research team on their positionality. We adopted an iterative process to Bacchi's WPR steps, sharing our reviews across the team, and revisiting our analyses against each step. The central themes were generated through reflexive cycles of coding, discussing and interpreting the data to generate themes which captured the core tenets of policy discourse. The analysis is presented here integrating Bacchi's approach, in line with other comparable analyses (e.g., Griffiths and Trebilcock, 2023; Richards-Gray, 2022; Whitelaw et al., 2022), with the seventh step (reflexivity) presented separately at the end of the findings.

## Findings

As detailed in Tables 1 and 2, we analysed three policy papers, six subsequent amendments, and 14 parliamentary debates related to the Immigration Health Surcharge.

The central themes of how the problem of migrants using healthcare services were constructed and represented were:

(i) Historic over-generosity toward transient populations,
(ii) A mechanism for fairness or inequity,
(iii) An unsustainable NHS,
(iv) The need for an infrastructure for enforcement.

## Historic over-generosity toward transient populations

A central problematisation and justification for implementing the Immigration Health Surcharge has been redressing the government's historic ‘over-generosity’ in allowing free access of NHS services to overseas visitors and migrants, who are assumed to have a short-term, transient, relationship with the UK:

‘The Government has set out in this consultation and elsewhere its view that our health system is overly generous to those with only a temporary relationship with the UK' (Department of Health, 2017: 7)

Mention of generosity also underpins parliamentary debates:

‘Use of the NHS by overseas visitors and migrants in England alone is estimated to cost up to £2 billion a year. Of this, the NHS spends nearly £1 billion a year on non-EEA temporary migrants *from* whom no cost is currently recovered […] The Government do not believe that the NHS should sustain this largesse.' (Hansard HL Debate, 10 March 2015). (Williams, 2015: Conservative)

Reference here to the term ‘largesse’ emphasises the generosity of the UK and by implying that migrants are exploiting this kindness, it positions the immigration charge as reasonable and appropriate. This is a formulation that simultaneously problematises the government (for its excessive generosity) and migrants (for having access to the NHS). Despite this apparent shared problematisation, the responsibility is placed with migrants to rectify supposed injustices through payment.

Temporality is used as a framing device to emphasise over-generosity. This is evident in both policy and parliamentary debates:

‘the NHS should not be free of charge to those with only a temporary relationship with the UK’, Making a Fair Contribution, 2017: 17)

‘It is right that we continue to prioritise the sustainability of the NHS and that temporary migrants make a financial contribution to NHS services available to them in the UK' (Hansard HL Debate, 12 December 2023). (Sharpe, 2023: Conservative)

Temporality is used in policy published in 2017 and in parliamentary debates in 2023, though there is an evident shift in emphasis to reflect the context of post-covid recovery, focusing on sustainability as described below. Short term migrants are constructed as a risk to the longevity of the NHS; in doing so protracted austerity is obscured in order to lay blame with migrants and therefore legitimise increasing the IHS.

Temporality is deployed to depict over-generosity towards people on student visas. The idea that temporary students should have access to the same privileges as permanent residents is positioned as unreasonable, hence situating the IHS as morally just:

‘They [students] say, "How can this be? Here we are in your country. If we were at home, we would be paying". They regard it as an absurd proposition. They are not here permanently and, in their view, they are therefore not entitled to the free care that those permanently resident in the UK should receive' (Hansard HL Debate, 28 November 2018). (Lansley, 2018: Conservative)

Migrants are situated as economic units, valued only through the level of long-held investment and sense of loyalty they demonstrate towards the country and the permanence of their relationship with it. The focus on ‘temporary migrants’ creates a distinction from ‘permanent migrants’ and in doing so initiates a deserving/undeserving dualism. Interestingly, some of the policy we reviewed actively prioritises enduring, historic, and ancestral familial connections. This is succinctly described in the announcement introducing the IHS:

The health surcharge will play a vital role in ensuring Britain's most cherished public service is provided on a basis that is fair to all who use it. For generations, the British public have paid their taxes to help make the NHS what it is today – the surcharge will mean temporary migrants will also pay their way. (UK Government, 2015)

The effect of this narrative is to problematise those who do not have a sense of historic pride and ownership. This engenders a casting of migrants and overseas visitors as illegitimately ‘taking’ from a system they have not contributed to in the long-term, again reinforcing over-generosity of the preceding system.

## A mechanism for fairness or inequity

The trope of the government's historic ‘over-generosity’ is underscored by several discursive strands that are both divisive and hostile. The problem constructed around over-generosity is intertwined with the idea of historic lack of fairness in the contributions made by migrants and overseas visitors when compared with British citizens, and that charging migrants resolves this and brings about necessary equity and fairness. Justice is a frequently occurring theme throughout the policies and debates, and the charging initiative is framed as a move towards fairness. In *Making a Fair Contribution* (2017), references to ‘*fair*’ and ‘*fairness*’ occurs 35 times, for example.

‘Our NHS is the envy of the world and we have no problem with overseas visitors using it - as long as they make a fair contribution, just as the British taxpayer does' (2015: 5)

The British public are situated as sole contributors to the NHS through their taxation*.* Taxpayer investment is framed as something the government needs to actively protect:

‘It is therefore our intention to make sure that only people living here and contributing financially to this country will get access to free NHS care' (Department of Health, 2017: 7)

This emphasis on financial contribution frames the NHS as a transactional consumerist endeavour, whereby only those paying into the service are afforded access (Bolton, 2002). This is somewhat removed from the founding ethos of the NHS to make healthcare accessible to all. Moreover, having historically been enabled by the government to access free healthcare, the advent of the surcharge sees a shift towards the representation of migrants as previously having shirked their responsibility, consequently acting as a financial drain on the economy and a burden on the NHS. This construction of unfairness is interwoven throughout the policy discourse, despite many migrants contributing to Britain through tax and national insurance, so that the additional charge of the IHS constitutes a *double taxation* and a *second* charge for migrants.

The notion of double taxation is visible in some of the parliamentary debates more critical of the IHS (and elsewhere e.g., Medact, 2020). Some MPs rallied against the surcharge for its fundamental *lack* of fairness. For instance, Sultana (2020) suggested that ‘*the NHS surcharge is a discriminatory double tax on migrants*’. This is reinforced by a member of the House of Lords, who said that:

‘The immigration health surcharge is not a fair contribution. The majority of migrants are taxpayers, so they will effectively be paying twice for any NHS treatment they receive: once through the IHS, and again through their taxes.' (Hansard HL Debate., 13 November 2018). (Khan, 2018: Labour)

The increased IHS and visa fees can be viewed as discriminatory practice because they directly target migrants, and act as a political apparatus and disciplinary mechanism to discourage migration. This is highlighted by the demonisation of migrants which has progressively become normalised:

‘We have become so hostile, so fast, that now such policies just seem normal. This change is a continuation of the Government's obsession with blaming all the country's problems on immigration. As a Green, I strongly resist any measure of hostility based on where in the world a person was born.' (Hansard HL Debate, 28 November 2018). (Jones, 2018: Green)

This position constructs migrants as political scapegoats, blamed and used to deflect political failings, resulting in discriminatory treatment towards migrants becoming socially acceptable. This simultaneously obfuscates the issue of protracted underfunding of the NHS. As Baroness Walmsley (Liberal Democrat) said: ‘*It ultimately serves to mask the main challenge facing the health service – a lack of cash*’ (Hansard HL Debate. 9 Feb 2017 (Walmsley, 2017)). Framing migrants as a burden on the NHS deflects political and ideological debate about underfunding.

Tropes of over-generosity and unfairness represent migrants as requiring substantial use of the NHS, despite running counter to the ‘healthy migrant effect’ (Kennedy et al., 2015). This is reinforced in parliamentary debates:

‘The evidence is very clear, particularly for people such as students, the younger population, that they have very little purchase on the NHS when they are here, because generally they are a healthier population' (Hansard HC Debate, 10 January 2024). (Hillier, 2024: Labour)

Further, migrants are also positioned in Parliamentary debates as contributing more than they receive:

‘They have put their own lives on the line, and continue to do so, to help us combat coronavirus. They have displayed the truly British qualities of commitment and stepping up to the mark in a time of need and crisis' (Hansard HL Debate, 23 September 2020). (Rosser, 2020: Labour)

Non-monetary contribution here is used to demonstrate the commitment of migrants to the country (and the injustice of the IHS), but it is an argument that does not only apply specifically to workers in health and social care. Although injustice is framed in relation to low utilisation, this fails to question the underpinning principle and ethics of charging migrants to access healthcare. Further, in the above quote, migrants are implicitly constituted as heroic, as a marker of their cultural assimilation into Britishness in the postcolonial era.

## An unsustainable NHS

Policies and debates overtly position the IHS as necessary for the longevity and sustainability of the NHS. The *Making a Fair Contribution* policy justifies charging as a mechanism to ensure the continuation of the NHS:

‘Whilst overseas visitors can access its services, in order for the NHS to be financially sustainable it is vital they make a fair contribution towards the cost of those services. […] The NHS is under increasing pressure and it is right that in the future everyone who benefits from its services makes a fair contribution to ensure it is sustainable and only those who are living here and contributing financially are entitled to receive free NHS care' (Department of Health, 2017: 7)

The problem formulation is a threat construction which suggests that ‘overseas visitors’ risk undermining the financial sustainability of the NHS. Therefore, the only way to prevent harm to the NHS is to implement charging. The implication of this, arguably hyperbolic, narrative is that the NHS will not be able to continue to function without this charge, which effectively locates legal migrants as burdening the NHS to such a degree that it is almost broken. This plays on sentimentality in the British psyche of the NHS as a much beloved institution (Seaton, 2023; Stewart, 2023) and, situating it at breaking point gives further galvanising public support for charging. Moreover, it draws on populist rhetoric implying that minority groups have benefitted from privileges and preferences (i.e., free healthcare) not available for free to the British taxpayer.

Sentiments of sustainability are also echoed in parliamentary debates where NHS revenue is constructed as a problem, with the IHS as a pathway to supporting sustainability:

‘We estimate that a further £220 million a year could be raised to support the NHS, helping to protect and sustain this country's world-class healthcare system for everyone who uses it' (Hansard HC Debate, 13 November 2018). (Nokes, 2018: Conservative)

The increases in the IHS fee (rising from £150 to £1035 annually) is framed as a reflection of the increasing costs in providing treatment, and situated in the context of post-Covid recovery. The accompanying narrative, however, changes across time. Originally framed as ‘good value for money’ is replaced with assertions about ‘the need for full cost’ recovery, demonstrating a hardening approach towards the treatment of migrants:

‘The health charge is designed to benefit our NHS and support its long-term sustainability. Our manifesto committed to increasing this charge to NHS cost recovery levels. The draft order delivers on that commitment, and I commend it to the Committee.' (Minister for Legal Migration and the Border, Hansard HC Deb., 10 January 2024). (Pursglove, 2024: Conservative)

The Impact Assessment for the 2020 IHS update (Home Office, 2020) explicitly names its benefits as reducing migration and improving social cohesion, confirming IHS as a device for creating a hostile environment for migrants and overseas visitors:

Other key non-monetised benefits by ‘main affected groups’. Lower immigration to the UK may result in some wider benefits (better social cohesion and reduced housing/transport congestion). Such impacts are expected to be small. Ensuring the surcharge is set at a level that reflects the average use of NHS services provided to those that pay it may increase public confidence in the immigration system. Revenue collected from the surcharge will be re-invested in the health service, ensuring its sustainability. (Home Office, 2023: 2)

Problem formulations relating to NHS sustainability and the framing of the NHS at breaking point as stimulus for the IHS becomes even more apparent with the suggestion that it will fund staff salaries:

‘We plan to increase the rates of the immigration health surcharge, which have been frozen for the past three years, despite high inflation and wider pressures facing the economy and the system in general, to ensure that it covers the full healthcare costs of those who pay it. Under our plans, the main rate will increase to £1,035, and the discounted rate for students and under-18s will increase to £776. That increase to the surcharge will help to fund the pay rise for doctors.' (Hansard HC Debate, 13 July 2023) (Glen, 2023: Conservative)

The proposition of using the IHS to meet rising salaries for doctors resulted in very little resistance from British taxpayers. Despite several public communications made by the Home Office (Glen, 2023; Home Office, 2020) suggesting direct transfer of IHS to the NHS, the income can be directed toward non-NHS organisations (Gower and McKinney, 2023). Responses to Parliamentary questions tabled in July 2022 state that ‘there is no central record of the proportion of IHS income received by NHS and non-NHS organisations’ (Kamall, 2022). These contrasting descriptions create ambiguity around how and where IHS is spent and how spending is monitored which, in turn, somewhat destabilises the rationale for the IHS, so strongly communicated (particularly in Making a Fair Contribution), that a fair contribution from migrants is necessary for the long-term sustainability of the NHS.

This problem construction is challenged in parliamentary debates, where the IHS itself is positioned as a threat to the sustainability of the NHS. Migration is positioned as fundamentally at odds with the interests of the NHS and the British public:

‘Labour's position on this modest increase in charging for our excellent health services in the UK is that it is in favour of more migration and less money for the NHS. Does he not realise how that will grate with the British public and his electors […]? We cannot fund an international health service.' (Hansard HC Debate, 13 November 2018). (Bridgen, 2018: Conservative)

Migrant workers are viewed as essential in meeting critical workforce shortages, and the IHS risks deterring overseas workers:

‘The UK is facing an NHS staffing crisis, and we desperately need to attract doctors and nurses from abroad, to at least plug short-term gaps' (Hansard HC Debate, 13 November 2018) (Khan, 2018: Labour)

Migrants are valued here only in terms of workforce utility; this silences intrinsic human and societal worth. The effect in policy debate is to create and sustain hierarchies by categorising and classifying migrants, with repercussions for those who are unable to demonstrate similar productivity.

Khan continues by highlighting the potential impact on migrant access to state services, alongside wider system level implications, he challenges assertions that the IHS is a mechanism for sustainability:

‘The immigration health surcharge goes hand in hand with the Government's hostile environment for migrants. Requiring them to pay such an exorbitant cost will push people to defer regularising their status. Without regularised status, a migrant cannot access housing, education and health services. This is not only a very difficult personal position, but poses a public health risk. In the end, the cost to the NHS will be greater, as people will not seek treatment until a problem reaches a crisis point' (Hansard HC Debate, Tuesday 13 November 2018) (*Khan, 2018*: Labour)

## The need for an infrastructure for enforcement

The WPR analysis also identified the problematisation of infrastructure needed for enforcing the IHS and recouping costs. This is despite the IHS being decided upon by the Secretary of State (2015a) and administered by the Home Office. The IHS must be paid before any visa application is made, and most received funds are allocated to relevant health departments (Gower and McKinney, 2023: 13). While there is no direct NHS involvement in the IHS, entitlements to NHS care need checking (McHale and Speakman, 2020). Infrastructure was visible through the ‘Immigration Health Charge Order’ (2015a), the ‘IHS Research Briefing’ (Gower and McKinney), and ‘Making a Fair Contribution’ (2017). The former two documents both imply implementation of the IHS requires the NHS to improve identification, monitoring, enforcement, and cost recovery processes of liable overseas visitors and migrants:

‘The NHS must get better at identifying patients who should be charged for their healthcare at an earlier stage of their treatment…[We are setting out our aim] to ensure that information on a person's eligibility for free healthcare is captured at their first point of contact with the NHS, regularly verified and available to other NHS organisations where necessary'. (Making a Fair Contribution, 2017: 5)

The phrase ‘regularly verified’ implies that people might not self-declare a change of circumstances, which constructs a view of migrants as untrustworthy. The policies articulate government plans to either draw on existing personnel (e.g., ‘entry clearance officer’ – Immigration Health Charge Order, 2015a, 2015b), recommission teams (e.g., the Cost Recovery Support Team), and/or to create new roles geared towards assisting delivery of the IHS programme:

‘NHS Improvement will also bring NHS overseas visitor and migrant cost recovery within its wider regulatory regime to drive up levels of senior engagement in Trusts. This will be achieved through […] working with the Department of Health to appoint National Clinical Champions for NHS overseas visitor and migrant cost recovery, who will promote and support delivery of the Programme amongst NHS clinicians and other frontline staff.' (Department of Health, 2017: 13)

Authors raise concerns regarding NHS staff being actively involved in immigration border control, and feeling under pressure to identify and make decisions on people's access to healthcare depending on their immigration status and IHS payment (McHale and Speakman, 2020; Nellums et al., 2018; Worthing et al., 2021).

Implementing charging has the potential to interfere with practices of care but as the government set out in *Making a Fair Contribution* (2017: 7), NHS staff should not become gatekeepers to healthcare provisions (‘*The role of NHS staff should not extend to immigration control, and clinicians should not be diverted from treating patients*’). Despite this sentiment, frontline staff and clinicians are framed as being in a prime position for implementing what might be deemed gatekeeping practices; identifying chargeable patients and blocking/barring treatment until payment is made:

‘[the NHS is required to] charge overseas visitors upfront and in full for any care not deemed by a clinician to be “immediately necessary” or “urgent” and/or cease providing such non-urgent care where payment is not received in advance of treatment beginning.' (Department of Health, 2017: 11)

Upfront payment positions and problematises migrants as posing a risk of non-payment. This is noted in the ‘Immigration Health Charge Order’ (2015a, 2015b) regarding the State's ability to ‘reduce, waive or refund all or part of a charge’, (Gower and McKinney, 2015a, p. 3), and cites the consequences of non-payment including a visa being ‘cancelled’, ‘revoked’, or rendered ‘invalid’ (ibid, p. 6).

## Positionality

Bacchi's seventh step asks the research team to be reflexive about their own position and bias. The team comprised individuals who currently (LLP, TS) or have previously (LF) lived as migrants, and where all team members are politically left leaning. The team are ideologically focused on equalities for marginalised people, and motivated to address the financial impact of terminal illness on migrants in the UK. Consequently, we came to the dataset with a bias in favour of migrants, not viewing them as a drain on UK systems.

## Discussion

This article has examined representations and problem formulations within policy documents and parliamentary debates related to the Immigration Health Surcharge. Our analysis showed strong alignment in how problems were framed across policy documents and debates. There were two key exceptions. First, although most of the dataset was awash with problematising migrants and overseas visitors, some parliamentary debates included a counternarrative highly critical of the IHS. Second, the parliamentary debates did not highlight the need to establish an infrastructure for enforcing the IHS.

Key problem formulations include the historic over-generosity of the government towards transient migrants. A deserving and undeserving dualism is constructed, with temporary migrants problematised for their insecure and impermanent relationship and contributions to the UK. Ensuring the long-term sustainability of the NHS is presented as a rationale for charging. In doing so, policies cast migrants as a risk to the country, further embedding a division between migrants and the British taxpayer. The counternarrative to this, debated by some MPs, frames the IHS as a double-charge for migrants whereby they pay both through the IHS and again through taxes.

Processes to monitor and recoup costs represent a normalisation of surveillance, disrupting a sense of privacy for migrants. They reinforce the link between healthcare provision and migration control and represent a clear implementation of welfare chauvinism (Guentner et al., 2016). This practice undermines medicine's ethical principles, moral obligations and approach to care (Reynolds and Mitchell, 2019). Indeed, involvement of healthcare in bordering presents an attempt to converge two completely contrasting agendas (Reynolds and Mitchell, 2019) and risks damaging the patient-doctor relationship (Kilner, 2014; McHale and Speakman, 2020). Yet while frontline staff are enlisted into identifying migrants’ NHS eligibility, workforce understanding and knowledge of the regulations and migrants’ entitlements is often lacking (Rassa et al., 2023). Although migrants are entitled to access urgent care and some other free services, in practice services are withheld through lack of knowledge of the regulations (Worthing et al., 2021) even for urgent health concerns (Nellums et al., 2018).

The problematisations of migrants within IHS policy and debate has important and immediate impacts. Patients fear that by simply accessing care and treatment they risk removal/deportation from the country or detrimental impacts to future visa applications (Bowling and Westenra, 2020; Griffiths and Trebilcock, 2023; Hacker et al., 2015; Joseph, 2017; McHale and Speakman, 2020). Bordering practices performed by NHS staff reproduce and disseminate these problematisations, resulting in dissuading migrants from seeking medical attention for fear of a focus not on medical needs but on immigration status (Medact, 2020). Hence, disciplinary mechanisms of fear and surveillance are employed to discourage migrants from accessing healthcare (Foucault, 1977). Migrants may therefore face delayed healthcare access, treatment, and diagnosis (Rassa et al., 2023), despite such access being framed in the 2025 immigration white paper as a marker of appropriate cohesion and integration (HM Government, 2025). Health staff who resist bordering practices seek to both question and disrupt the problematisation (Reynolds and Mitchell, 2019).

Framings of the IHS draw on historical narratives of what migrants contribute to British society. The historical context and genealogy draws from the broader socio-political context of prolonged austerity, the EU referendum, Brexit, the pandemic, and the cost-of-living crisis (Richards-Gray, 2022). Such discourses focus on what migrants’ ‘offer’ to the UK, and this rests on an economics lens and locates migrants as economic units, valued through their contribution. Anti-immigrant sentiment runs rife through public life driven by political agendas and parties, as it has recently been seen in the recent riots in the UK (Boukari and Devakumar, 2024).

The emphasis on contribution is born from the NHS system which is founded on the basis of people paying for it prospectively in their taxes which, consequently, ‘“forces” policy actors to think about the circumstances under which free care should be provided to people who have not paid prospectively’ (Grit et al., 2012: 60–61). The problem is produced, disseminated and defended through subsequent legislation and healthcare practices. Yet, some authors argue that ‘[a]ccess to the NHS does not and should not rely on the scale of individual contribution, through tax, national insurance, or any other means’ (Medact, 2020, p.11). Focusing on contributions risks decentralising people's human right to the highest attainable standard of healthcare as underpinned by the International Covenant on Economic, Social and Cultural Rights (Article 12) (Ministry of Justice, 2022), an interesting omission given the focus on justice, ‘fairness’, and ‘equity’ embedded in IHS policies. The dominant deployment of an economics lens also has wider symbolic ramifications for migrants who are not working, including those who are terminally ill and unable to, and for whom financial precarity and destitution may be a stark reality (Fitzpatrick et al., 2023; Page, 2023; Ramachandran, 2024).

## Conclusion

Despite their important role in British civil society and in delivering critical health and social care services, the WPR analysis shows that the overwhelming frame of migrants is to see them as a risk to the National Health Service. IHS is an ideological project which reflects and reinforces prevailing negative narratives about migrants in the UK.

Although a small number of parliamentary debates offered critical counter-perspectives – highlighting double-charging, ethical concerns, and the erosion of healthcare rights – these were exceptions within a wider context of consensus. The institutionalisation of surveillance and bordering within healthcare settings normalises the treatment of migrants as risks to be managed, rather than patients to be cared for. This convergence of healthcare and immigration control threatens foundational medical ethics and compromises migrants’ access to timely and adequate care.

The framing of healthcare access through economic contribution not only marginalises those unable to work but also undermines human rights-based approaches to health. The IHS emerges as a product of broader socio-political forces – economic austerity, anti-immigrant sentiment, and nationalism – which together shape who is deemed worthy of care. Recognising the historical and ideological underpinnings of these framings is essential to challenging the inequities embedded in current healthcare and immigration policy. Challenging the problematisation of migrants would centralise a reframing on rights, ethics, and social solidarity. We believe this is urgently needed to counteract the exclusionary and punitive logics of the IHS.

## Appendix Table 1: search terms

**Table:**

Search Engine	Search Terms
Google	Migrants access to healthcare UK; Immigrants and access to healthcare; Migrants access to healthcare policy UK; NHS migrants access to healthcare Immigration health surcharge; NHS healthcare for migrants.
Gov.UK	Search terms: Migrants Topic: Health and social care Content type: Policy papers and consultations; Guidance and Regulation; Policy papers and consultations; Guidance and Regulation; Search terms: Immigration / immigrants Topic: Health and social care Content type: Policy papers and consultations; Guidance and Regulation; Search terms: work visas /employment visas Topic: Health and social care Content type: Guidance and Regulation; Policy papers and consultations.
NICE/House of Commons Library	Immigration/Immigrants; Migration/migrant; Migrants access to healthcare; Immigration Health Surcharge.
